# Investigating the Cycling Stability of Fe_2_WO_6_ Pseudocapacitive Electrode Materials

**DOI:** 10.3390/nano11061405

**Published:** 2021-05-26

**Authors:** Julio César Espinosa-Angeles, Nicolas Goubard-Bretesché, Eric Quarez, Christophe Payen, Moulay-Tahar Sougrati, Olivier Crosnier, Thierry Brousse

**Affiliations:** 1 Institut des Matériaux Jean Rouxel, Université de Nantes, CNRS, IMN, F-44000 Nantes, France; julio.espinosa@cnrs-imn.fr (J.C.E.-A.); nicolas.goubard@cnrs-imn.fr (N.G.-B.); eric.quarez@cnrs-imn.fr (E.Q.); christophe.payen@cnrs-imn.fr (C.P.); thierry.brousse@univ-nantes.fr (T.B.); 2 Réseau sur le Stockage Electrochimique de l’énergie (RS2E), FR CNRS 3459, CEDEX, 80039 Amiens, France; moulay-tahar.sougrati@umontpellier.fr; 3 Institut Charles Gerhardt Montpellier (ICGM), UMR 5253, Université de Montpellier, 34095 Montpellier, France

**Keywords:** Fe_2_WO_6_, electrochemical capacitor, pseudocapacitance, Mössbauer spectroscopy

## Abstract

The stability upon cycling of Fe_2_WO_6_ used as a negative electrode material for electrochemical capacitors was investigated. The material was synthesized using low temperature conditions for the first time (220 °C). The electrochemical study of Fe_2_WO_6_ in a 5 M LiNO_3_ aqueous electrolyte led to a specific and volumetric capacitance of 38 F g^−1^ and 240 F cm^−3^ when cycled at 2 mV·s^−1^, respectively, associated with a minor capacitance loss after 10,000 cycles. In order to investigate this very good cycling stability, both surface and bulk characterization techniques (such as Transmission Electron Microscopy, Mössbauer spectroscopy, and magnetization measurements) were used. Only a slight disordering of the Fe^3+^ cations was observed in the structure, explaining the good stability of the Fe_2_WO_6_ upon cycling. This study adds another pseudocapacitive material to the short list of compounds that exhibit such a behavior up to now.

## 1. Introduction

Electrochemical capacitors (ECs) are energy storage devices with high power density and moderate energy density, which makes them interesting devices to be coupled with batteries [1,2]. Most of the current commercial ECs are based on activated carbon electrodes that exhibit specific capacitance in the range 100–200 F g^−1^ depending on the electrolyte, the pore size distribution, and other parameters [3,4]. They are known as Electric Double Layer Capacitors (EDLCs) and present relatively low volumetric performance due to the low density of the active material (~1 g·cm^−3^). One characteristic of EDLCs is the charge storage mechanism, which processes mainly by electrostatic interactions at the carbon/electrolyte interface, leading to the well-known rectangular signature on the related cyclic voltammograms (CV) [5,6]. Some transition metal oxides and nitrides are also exhibiting such capacitive-like signature [7,8] with a similar electrochemical behavior. However, the charge storage mechanism in these latter compounds relies upon fast and reversible redox reactions happening at the vicinity of the electrode material, as it has been already demonstrated for several materials such as RuO_2_ [9], MnO_2_ [10,11], or Fe_2_O_3_ [12]. Such a mechanism is reported in the literature as pseudocapacitance, due to the electrochemical shape of the CV [1], but which originates from a different charge storage mechanism than for EDLC electrode materials. This pseudocapacitive behavior depends mainly on the existence of specific electroactive cations in the electrode materials. Moreover, some other parameters such as the specific surface area, the morphology, and the accessibility of active sites are also important since it remains a surface reaction that occurs at the electrode/electrolyte interface [13].

The family of Fe-W-O has been studied in our team in the wolframite phase FeWO_4_, showing interesting performance as an electrode material for EC operated in a neutral aqueous electrolyte [14,15]. The role of the Fe^3+^/Fe^2+^ redox couple has been recently proven to be responsible of the pseudocapacitive behavior [16]. Apart from its pseudocapacitive behavior, this compound is interesting to prepare high volumetric capacitance electrode due to its high density compared to carbon compounds. Thus, from fundamental and applied point of views, the Fe-W-O system was further investigated with the synthesis of Fe_2_WO_6_. Unlike FeWO_4_, this ternary oxide contains only Fe^3+^ in the crystal structure. When synthesized at high temperature (>950 °C), the structure of Fe_2_WO_6_ (γ polymorph) can be described with two different zigzag chains, one containing only Fe(1) cations and the other containing Fe(2) and W cations being alternated each other [17,18]. Different studies have reported the properties of Fe_2_WO_6_ as a negative electrode for lithium-ion batteries [19], photocatalyst [20] and photoelectrode materials [21]. However, Fe_2_WO_6_ has only been synthesized under high temperature synthesis conditions, leading to a large mean particle size and low specific surface area, which are detrimental to surface reactions such as for a pseudocapacitive behavior. In this regard, we report here a polyol method for the synthesis of Fe_2_WO_6_ at low temperature (220 °C), thus limiting the growth of the particles [22,23]. Although, only Fe^3+^ is present in Fe_2_WO_6_, a very similar electrochemical behavior as for FeWO_4_ has been observed with similar specific capacitance values and only a slight capacitance fade after cycling (85% capacitance remaining after 10,000 cycles). We investigated the possible changes occurring in this electrode material caused by a long-term cycling using several characterization techniques 

## 2. Materials and Methods

### 2.1. Polyol-Mediated Synthesis

Fe_2_WO_6_ was synthesized for the first time following the reported polyol-mediated synthesis of several nanoscale transition metal tungstates, such as FeWO_4_ [14,22]. First, 50 mL of diethylene glycol, DEG 99% (HOCH_2_CH_2_)_2_O (Alfa Aesar, Thermo Fisher, Illkirch, France), was heated up to 80 °C to dissolve 10.55 mmol of iron (III) nitrate nonahydrate, Fe(NO_3_)_3_·9H_2_O 98% (Alfa Aesar, Thermo Fisher, Illkirch, France), under vigorous stirring until acquiring a homogeneous solution. Then, an aqueous solution of 5 mmol sodium tungstate dihydrate, Na_2_WO_4_·2H_2_O 99% (Acros Organics, Thermo Fisher, Illkirch, France), was added dropwise and directly thereafter the pH was very carefully adjusted to 6 using a solution of ammonium hydroxide, NH_4_OH 28% (Alfa Aesar, Thermo Fisher, Illkirch, France), resulting in a reddish suspension-like. Once the pH was stabilized, the mixture was heated up to 220 °C and kept for 1 h until a complete nucleation occurs. After natural cooling to room temperature, the particles were purified by centrifugation (20 min, 10,000 rpm) and suspended in ethanol, glacial acetic acid, and distilled water three times in order to remove all the remaining salts and other side products of the reaction. The obtained powder was then dried at 60 °C for 24 h in air and grinded prior to several heat treatments under air at different temperatures, ranging from 400 °C up to 800 °C.

### 2.2. Electrode Preparation

The composite electrodes were prepared in agreement to that described by Brousse et al. [24]. The electrode composition was as follows: 60%wt of Fe_2_WO_6_ as the active material, 30%wt of conductive carbon (Carbon Black by Superior Graphite, Chicago, IL, USA) to improve electronic conductivity and 10%wt of binder, Polytetrafluoroethylene (PTFE Sigma Aldrich, Merck KGaA, Darmstadt, Germany), to ensure the mechanical strength of the electrode. The mixture was suspended in ethanol and heated up to 60 °C under vigorous stirring until the complete evaporation of ethanol. The black remaining slurry was then cold-rolled until reaching a composite film with a thickness between 100 and 150 μm. After drying at 60 °C to remove the remaining ethanol, 12 mm diameter disks were cut out and pressed at 900 MPa onto stainless steel or titanium grids, used as current collectors. The mass loading of the prepared electrodes varied from 5 to 10 mg cm^−2^, which is an accepted loading for assessing the properties of an electrode material used in electrochemical capacitors [25].

### 2.3. Characterization of the Material and Electrodes

The crystallographic structure of the different samples was investigated by X-ray diffraction (XRD) using a PANalytical X’Pert Pro diffractometer (Malvern Panalytical, Almelo, the Netherlands) with an X’Celerator detector and Cu-*Kα*1-*Kα*2 (*λ* = 1.54060, 1.54439 Å) radiation. The acceleration voltage and current were 40 kV and 40 mA, respectively. The diffraction patterns were collected in the 2θ range of 10 to 70° and a step scan of 0.0167°. For the heat treatment study, a high temperature chamber XRK 900 (Anton Paar, Les Ulis, France) was used under air with a heating rate of 6 °C/min. The chemical analysis was obtained with EDX (by Oxford Instruments NanoAnalysis, High Wycombe, United Kingdom), X-Max, detector 50 mm^2^ installed on a Merlin SEM (from Carl Zeiss, Oberkochen, Germany). The specific surface area of the powders was determined using the BET method (Brunauer-Emmett-Teller) from the 77 K nitrogen adsorption curves with a Quantachrome Nova 4200e equipment (Anton Paar, Les Ulis, France).

The microstructure, surface study of the electrode samples, morphology and particle size were studied with a transmission electron microscope ThermoFisher S/TEM Themis G3 at 300 kV (Breda, The Netherlands) point to point resolution: 0.18 nm.

^57^Fe Mössbauer spectra were measured in transmission mode with a ^57^CoRh source. During the measurements, both the source and the absorber were kept at ambient temperature (294 K). The spectrometer was operated in the constant acceleration mode with a triangular velocity waveform. The velocity scale was calibrated with the magnetically split sextet spectrum of a high-purity α-Fe reference absorber at room temperature. Since the fitting of the data is not possible using one doublet (one environment), they were fitted with an appropriate distribution of 6 Lorentzian profiles representing quadrupole doublets by the least-squares methods using the program PC-Mos II (Version 1.0 by FASTComTec, Oberhaching, Germany) [26]. In this way, spectral parameters such as the quadrupole splitting (QS), the isomer shift (IS), the linewidth at half maximum (LW) of the different spectral components were determined. Isomer shifts are given relative to the α-Fe.

The magnetization experiments were performed using a commercial SQUID magnetometer (MPMS-XL7 by Quantum Design, San Diedo, CA, USA) from 5 to 300 K at a field strength of H = 200 Oe. The applied field value was the same as the one used by Guskos et al. [18].

The electrochemical performance of all the samples was performed by cyclic voltammetry with a VMP3 galvanostat–potentiostat (from Biologic run under ECLab software (version V11.36, Seyssinet-Pariset, France). The experiments were conducted using a three-electrode electrochemical setup employing Ag/AgCl (3M NaCl) as the reference electrode and a platinum grid as the counter electrode. A neutral 5M LiNO_3_ aqueous solution was used as the electrolyte, and all the experiments were performed in a [−0.6 V; 0 V] vs. Ag/AgCl potential window.

## 3. Results and Discussion

### 3.1. Synthesis and Structural Characterization

**Figure 1:**
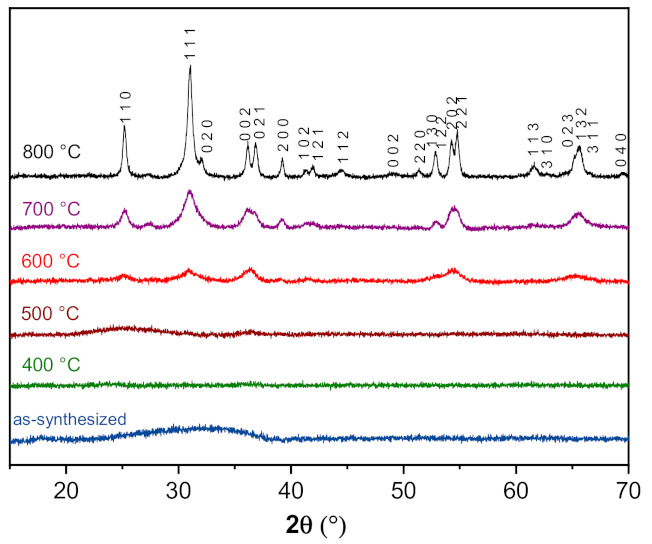
Evolution of the XRD patterns of Fe_2_WO_6_ synthesized by polyol method from 30 °C up to 800 °C.

Surprisingly, the room-temperature polyol-mediated route used for the synthesis of Fe_2_WO_6_ leads to an amorphous material, unlike some of the other tungstates synthesized by a similar method and already reported in the literature [14,22]. An X-ray diffraction study enabled us to follow the crystallization of the powder while increasing the temperature. Figure 1 shows a selection of the diffractograms collected at different temperatures. As for the material synthesized at room temperature, an amorphous phase is still observable when the temperature reaches 400 °C. Some broad features begin to grow at 600 °C, resulting in a well crystallized pattern at 800 °C. The cell parameters of this latter sample can be refined in a *Pbcn* space group with *a* = 4.5995(15) Å, *b* = 5.6043(19) Å, *c* = 4.9718(19) Å (see supplemental Table S1 and Figure S1 [27]), with both Fe and W atoms occupying the same crystallographic site with a 2:1 ratio. Although this random distribution of Fe and W in the same site does not significantly affect the resulting Bragg positions, this peculiar structure will be depicted in a forthcoming paper.

The as-synthesized sample (without any further heat treatment), and the samples heat treated at 400 °C, 600 °C, and 800 °C were selected to follow some extra characterizations and will be referenced as FWO-RT, FWO-400, FWO-600, and FWO-800, respectively, in the text. All samples kept a Fe/W ratio ~ 2:1 according to the EDX measurements. The BET results showed a decrease in the specific surface area (SSA) while increasing the temperature, being 133, 121, 26, and 11 m^2^ g^−1^, for FWO-RT, FWO-400, FWO-600, and FWO-800, respectively. The electrochemical behavior of these selected samples has been investigated by cyclic voltammetry of composite electrodes in aqueous 5M LiNO_3_ electrolyte.

### 3.2. Electrochemical Characterization

**Figure 2:**
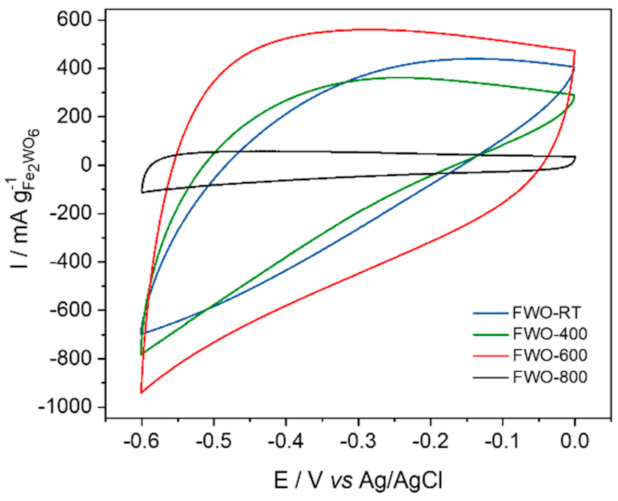
Cyclic voltammograms of the samples FWO-RT, FWO-400, FWO-600 and FWO-800 at 20 mV s^−1^ in 5M LiNO_3_ from 0 to −0.6 V vs. Ag/AgCl.

Figure 2 shows the cyclic voltammogram recorded at 20 mV·s^−1^ for the four Fe_2_WO_6_ samples selected out of the annealing study. The materials were studied in composite electrodes in 5M LiNO_3_ aqueous solution (pH~7) in a three-electrode setup from 0 to −0.6 V vs. Ag/AgCl. For all the materials, no redox peaks can be observed, thus ruling out a faradic response for the electrode materials. Instead, a distorted rectangular shape is observed for all the materials but with significant differences. It can be noticed that the low temperature samples FWO-RT and FWO-400 present a “rugby ball” shape voltammogram, corresponding to a very resistive composite electrode, with a specific capacitance of 17 F·g^−1^ and 15 F·g^−1^, respectively. The high temperature sample FWO-800 exhibits a more rectangular shape typical of pseudocapacitive oxides [28,29] but with a very low capacitance (3 F·g^-1^). On the other hand, the sample FWO-600 exhibits a quasi-rectangular shape CV with a specific capacitance of 23 F·g^−1^ at 20 mV·s^−1^. Such a behavior is very similar to that observed for FeWO_4_ material cycled under the same condition, [15,16], which has been attributed to the Fe^3+^/Fe^2+^ redox couple [16].

**Figure 3:**
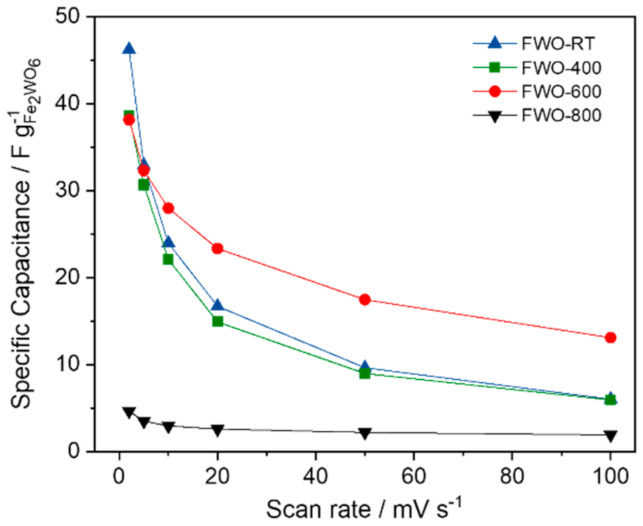
Evolution of the specific capacitance in F g^−1^ vs. scan rate in 5M LiNO_3_ for the selected Fe_2_WO_6_ samples.

**Figure 4:**
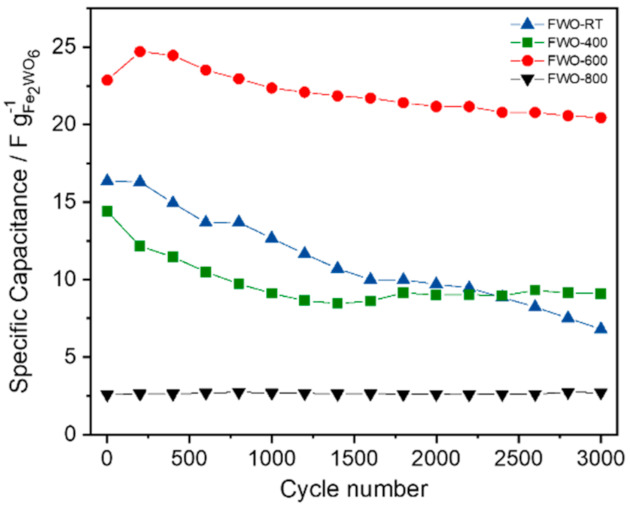
Cycling stability up to 3000 cycles at 20 mV s^−1^ in 5M LiNO_3_ for the selected Fe_2_WO_6_ samples.

Figure 3 displays the values of the specific capacitance in Farads per gram (F g^−1^) at different scan rates for all the samples. It is observed that despite a capacitance value of 47 F g^−1^ for the FWO-RT sample when cycled at 2 mV s^−1^, a rapid fade occurs while increasing the scan rate. Sample FWO-400 also exhibits a similar behavior while FWO-600 keeps the best performance even at 100 mV s^−1^. It can be noted that for this latter sample the specific capacitances measured at the different scan rates are within the same range as those measured for FeWO_4_ [13]. Up to 38 F g^−1^ can be obtained, corresponding to a volumetric capacitance of 240 F cm^−^^3^ when cycled at 2 mV s^−1^. This latter value was calculated considering a density of 6.3 g cm^−^^3^, measured by helium pycnometry. Such an interesting density can be exploited to implement this oxide as a high volumetric capacitance electrode in aqueous based asymmetric devices. The electrochemical stability of all the samples was then studied over 3000 cycles at 20 mV s^−1^. Figure 4 shows that after a slight increase during the first 200 cycles for FWO-600, almost 90 % of capacitance retention can be achieved after 3000 cycles, which is seldomly reported for pseudocapacitive electrodes. This slight increase has already been observed in our lab for similar materials, due to the impregnation time with the aqueous electrolyte throughout the porosity of the composite electrode. For FWO-400 and FWO-RT samples, a quicker fade in capacitance can be observed with only 65% and 50% capacitance retention, respectively. Finally, the FWO-800 exhibits a stable but very low capacitance upon the 3000 cycles.

Clearly, a compromise between the crystallinity and the specific surface area (or the particle size) has a direct impact on the electrochemical performance of the material [14,30] in order to get good capacitance values associated with both interesting cycling stability and power capability. The low temperature sample presents the highest SSA (up to 133 m^2^ g^−1^) compared to FWO-600 (26 m^2^ g^−1^), however, this latter sample achieves the best performance. Heating at 600 °C allowed obtaining a relatively high specific surface area in order to maintain a high capacity, while avoiding a progressive dissolution upon cycling as it is observed for the amorphous materials, as-prepared, or sintered at lower temperatures.

### 3.3. Long Cycling Stability Study

**Figure 5:**
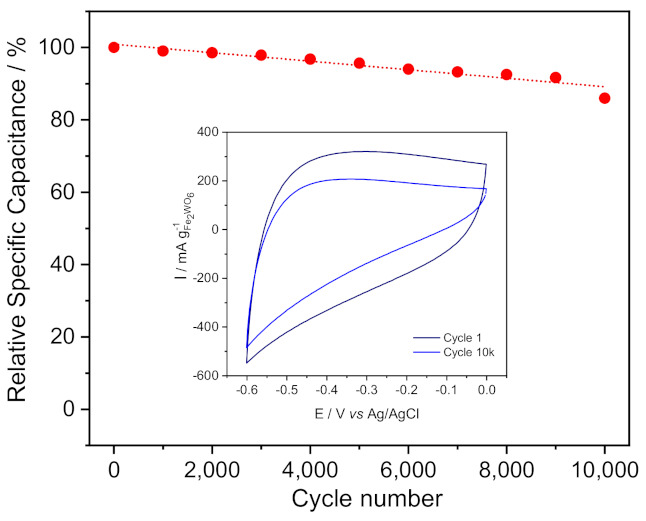
Long term cycling stability for FWO-600 sample at 20 mV s^−1^ in 5M LiNO_3_. Inset: cyclic voltammograms at cycle 1 and at cycle 10,000.

The changes occurring upon a long-term cycling of pseudocapacitive electrodes are scarcely investigated [30,31]. In order to get further insights into the structural or/and microstructural modifications occurring to the materials upon a long-term cycling, we performed more in-depth analyses. Thus, a FWO-600 electrode was selected for such purposes since it exhibits the best electrochemical performance in terms of capacitance, power capability and cycling stability. The material was submitted to 10,000 cycles (charge/discharge) at a cycling rate of 20 mV s^−1^. Figure 5 shows the relative capacitance after the long cycling experiment with the inset presenting the first and last cyclic voltammograms. The remaining capacitance after 10,000 cycles is close to 85%, with a very similar CV shape compared to the first cycle (inset Figure 5). No obvious degradation of the electrode nor material dissolution can be observed in the electrochemical cell.

As a comparison, N.L. Wu et al. [29] assigned the fade in capacitance of a MnO_2_ electrode in aqueous 1M KCl to several factors, including severe surface modifications induced by varying the upper cut-off potential. In order to fully investigate the changes that could occur to Fe_2_WO_6_ particles after an extended cycling, several electrodes (prior cycling, after 10,000 cycles at 20 mV s^−1^, and a “blank” electrode soaked in the electrolyte but without cycling) were cut into small pieces to perform experiments using different characterization techniques on the exact same material for all the experimentations. 

#### 3.3.1. Surface Observation by Transmission Electron Microscopy

**Figure 6:**
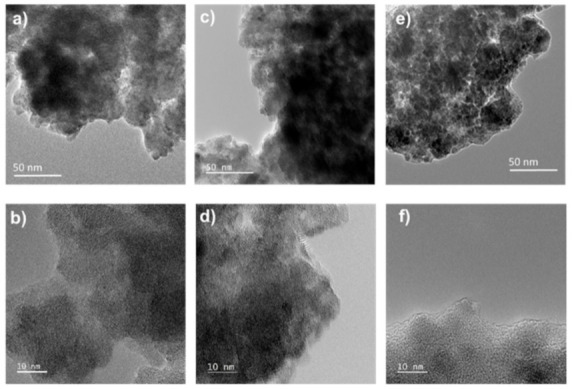
TEM images of raw FWO-600 particles (**a**,**b**), after 10,000 cycles (**c**,**d**) and from the blank electrode soaked in the electrolyte (**e**,**f**). Scale bar for figures a, c and e is 50 nm while it is 10 nm for figures (**b**,**d**,**f**).

First, the possible surface changes were investigated using TEM observations. This study was performed by comparing a series of three different electrodes: a piece of dry and non-cycled FWO-600 composite electrode (Electrode film, Figure 6a,b), FWO-600 electrode cycled 10,000 times (Electrode 10k, Figure 6c,d) and a FWO-600 electrode that was not cycled but only soaked in the electrolyte (Electrode blank, Figure 6e,f) for the same period of time as the electrode cycled 10,000 times. At low magnification (Figure 6a,c,e), all the samples present similar aggregates with an average size close to 100 nm, composed of very small irregular shape primary particles. From the TEM images it is difficult to claim significant particle size distribution in the different samples. At higher magnification, some small crystalline domains of a few nm can even be observed, without relevant differences between each other. These observations are in good agreement with the broad peaks shown on the XRD patterns (Figure 1). Interestingly, for all the samples, clear and sharp edges are visible at the surface of the particles. No dissolution or formation of extra layers or coatings can be detected, contrary to what can be observed for MnO_2_ after extended cycling [32]. Similarly, the particles extracted from the “Electrode blank” presented no distinguishable effect. Thus, neither cycling nor electrolyte interaction induce significant and visible changes at the surface of FWO-600 particles.

Microstructural changes do not seem to occur upon cycling or prolonged exposure to the electrolyte, and the small capacitance fade observed after 10,000 cycles must be investigated with more specific analytical tools that could evidence such modification.

#### 3.3.2. Mössbauer Spectroscopy Analysis

**Figure 7:**
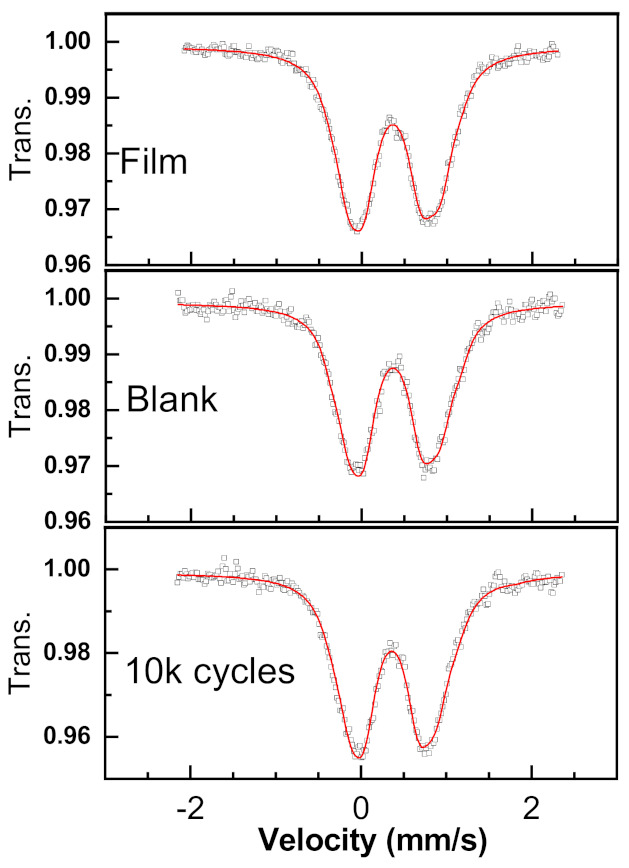
Room temperature Mössbauer spectra for non-cycled FWO-600 electrode (“Film”), non-cycled but soaked in electrolyte (“Blank”) and cycled electrode after 10,000 cycles at 20 mV s^−1^ (“10k cycles”).

Mössbauer spectroscopy is perfectly adapted for the study of Fe-based materials, as it should enable to reveal several modifications of Fe cations in the Fe_2_WO_6_ structure such as their oxidation state and environment. Subsequently, ^57^Fe spectra were collected at room temperature on the same samples that were used for the TEM observations. Mössbauer spectra of the as prepared electrode, after being soaked in the electrolyte (blank) and after 10,000 cycles are shown in Figure 7. The as prepared electrode spectrum agrees well with the data reported by Birchall et al. for Fe_2_WO_6_ powder [33]. The fitting was performed with an appropriate distribution of 6 Lorentzian profiles representing quadrupole doublets by least-squares methods (Figure S2). All the iron is in a ferric state and in high spin octahedral configuration. The obtained average isomer shifts and quadrupole splitting (given in Table 1) show that no Fe^2+^ can be detected in all the samples, thus confirming that the low temperature synthesis of Fe_2_WO_6_ leads to a pure phase. Moreover, no significant change is observed after 10,000 cycles or after prolonged soaking in the electrolyte, thus discarding any irreversible formation of Fe^2+^ upon cycling or soaking. Very tiny differences can be observed in terms of QS distribution (Figure S3) showing a slight increase of the weight of high QS contribution (around 1.2 to 1.5 mm/s). This might indicate a possible but very low increase of the iron sites distortion after cycling or soaking in the electrolyte. The experimental data do not exclude the formation of some superparamagnetic iron hydroxides [34], even if the magnetization investigation (3.1.3) does not support the presence of such phases.

Therefore, both accurate TEM observation of the surface of the FWO-600 material and Mössbauer spectroscopy experiments performed before and after cycling do not allow us to explain the capacitance fade observed after 10,000 cycles. No significant change can be observed concerning the surface morphology and on the Fe atoms environment. The possible changes after cycling were also investigated on the same electrodes regarding the magnetization properties of the FWO-600 sample.

#### 3.3.3. Magnetization Measurements on FWO-600 Electrodes

As for the previous experiments, magnetization measurements were performed on Electrode film, Electrode 10,000, and Electrode blank samples. Measuring conditions were the same as those used by Guskos et al. [18] for studying pristine Fe_2_WO_6_. Each sample was measured on warming two times: once when the sample had been cooled in the absence of a field (zero-field-cooled, ZFC) and once when the sample had been cooled in the measuring field of 200 Oe (field-cooled, FC). Magnetizations of the mixture of conductive carbon black and PTFE binder and of the Fe_2_WO_6_ polyol powder were also measured separately. The mass magnetization of the mixture of carbon plus PTFE was found to be very small, and the data for pristine Fe_2_WO_6_ were consistent with those published in reference [18].

**Figure 8:**
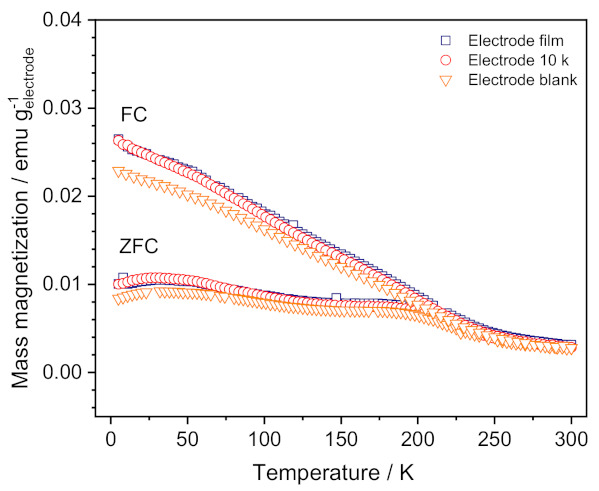
T-dependent FC (field-cooled) and ZFC (zero-field-cooled) mass magnetizations for non-cycled “Electrode film”, cycled electrode “Electrode 10,000”, and non-cycled but soaked in electrolyte “Electrode blank”.

Figure 8 presents the T-dependent FC and ZFC mass magnetizations for the three electrodes studied. Data for the electrode film and for the electrode blank, which was only soaked in electrolyte but without being cycled, are very similar to those obtained for pristine Fe_2_WO_6_, in that both the ZFC-FC bifurcation at about 250 K and the ZFC step-like anomaly at about 200 K are clearly visible [18]. As can be seen in Figure 8, these two latter features, which are a hallmark of crystallized Fe_2_WO_6_, are still observed in the T-dependent magnetization of the 10 k cycled electrode. Therefore, our data indicate that neither soaking nor cycling induce qualitative change in the bulk magnetic behavior of Fe_2_WO_6_. In other words, the bulk of the oxide powder remains crystallized and there is no change in the chemical composition of the bulk upon cycling. However, it does not preclude chemical and/or structural changes on the surface of the oxide as these bulk magnetization measurements do not probe surfaces.

Consequently, the magnetization measurements performed on the different samples tend to indicate that there is no amorphization nor modification of the iron cations environment upon cycling the electrode. The magnetic properties of the Fe_2_WO_6_ material are therefore not affected by long-term cycling in the 5M LiNO_3_ aqueous electrolyte.

## 4. Conclusions

In this study, Fe_2_WO_6_ was synthesized for the first time at low temperature (220 °C) using a polyol-mediated route. The amorphous as-synthesized oxide powder was further annealed in air at different temperatures, showing the evolution of the crystallization starting at 600 °C, and leading to a well-crystallized material at 800 °C. Simultaneously, the specific surface area decreased upon increasing the temperature. The electrochemical performance and cycling stability of selected Fe_2_WO_6_ powders was investigated as negative electrode materials in an electrochemical capacitor using a 5M LiNO_3_ electrolyte. The best performance was achieved with the sample annealed at 600 °C (FWO-600), which also exhibited the longest cycling stability. A specific and volumetric capacitance of 38 F g^−^^1^ and 240 F cm^−^^3^ were obtained when cycled at 2 mV·s^−1^, respectively. A quasi-rectangular shape corresponding to a typical pseudocapacitive signature was depicted and strong similarities with previously studied FeWO_4_ were observed. 

An interesting long-term stability (up to 10,000 cycles at 20 mV s^−1^) was observed for FWO-600 with only 15% loss in capacitance. TEM observations, Mössbauer spectroscopy, and magnetization measurements were further used in order to correlate the fade in capacitance to microstructural or structural changes. All the experiments demonstrated that the morphology, crystallographic structure, and magnetic properties were not affected by the electrochemical cycling nor by a simple prolonged soaking in the electrolyte. No partial dissolution at the surface of the particles, nor Fe^2+^ formation can be detected, confirming the stability of the material. However, some slight corrosion of the current collector at the interface with the electrode was observed, which could be due to some dissolved oxygen in the aqueous electrolyte, thus explaining the capacitance fade after 10,000 cycles. Thus, Fe_2_WO_6_ represents another multicationic oxide that exhibits interesting pseudocapacitive performance in terms of density, volumetric capacitance and long-term cycling behavior. Moreover, this material can therefore be of interest for assembling a full asymmetric electrochemical capacitor with a neutral aqueous electrolyte, associating Fe_2_WO_6_ with, for example, MnO_2_ as both the negative and positive electrode materials, respectively. 

These results bring some new highlights about the mechanism ruling stability of multicationic oxides upon cycling. Adjusting different parameters such as the crystallinity, microstructure or specific surface area of the electrode material could lead to optimized electrochemical properties of oxide materials used in aqueous-based electrochemical capacitors. 

## Figures and Tables

**Table 1:** Room temperature ^57^Fe Mössbauer hyperfine parameters, <IS>, <QS> and <LW> are the average isomer shifts, quadrupole splitting and linewidth respectively.

Sample	<IS> (mm/s)	<QS> (mm/s)	<LW> (mm/s)
Film	0.37	0.85	0.38
Blank	0.39	0.84	0.35
10k cycles	0.40	0.79	0.36

## Supplementary Materials

The following are available online at https://www.mdpi.com/article/10.3390/nano11061405/s1, Table S1: Atomic coordinates and atomic displacement parameters of Fe_2_WO_6_ polyol sample at 800°C with *a* = 4.5995(15) Å, *b* = 5.6043(19) Å, *c* = 4.9718(19) Å, *V* = 128.16(8) Å^3^, SG *Pbcn*. Figure S1: Final Rietveld refinement plot of the XRD data at 800°C of the Fe_2_WO_6_ polyol sample with Cu-*Kα*1*-Kα*2 radiation (R(obs) = 3.26 %, GOF = 1.33, wRp = 5.61 %). The inset shows the hypothetical structure of Fe_2_WO_6_, which can be described as the stacking along the *a* axis of layers made of zigzag chains of edge-sharing Fe/W octahedra shifted from one layer to another with a (1/2, 1/2, 0) vector. Layers are connected to each other through corner sharing octahedra. Figure S2: Room temperature ^57^Fe Mössbauer spectra of all the samples showing, as described in the text. Figure S3: QS distribution for the three samples.
