# Supplementary material for: Investigating the Cycling Stability of Fe2WO6 Pseudocapacitive Electrode Materials
Source: Nanomaterials (Basel). 2021 May 26;11(6):1405. doi: 10.3390/nano11061405 (PMC8230245; doi:10.3390/nano11061405)
Supplement: Supplementary file 1 [file nanomaterials-11-01405-s001.zip › nanomaterials-1224625-supplementary.pdf]

Supplementary Information

1) Rietveld analysis of the XRD data was performed using JANA 2006.[1]

The atomic coordinates and atomic displacement parameters are reported in Table S1. The reflections observed in the X-ray powder pattern can be indexed in a small unit cell with *a* axis equal to one-third that of the α-Fe_2WO_6, the low temperature form of Fe_2WO_6 with the columbite structure.[2] It is as if the structure adopts the α-PbO_2 structure without cation ordering and has a single mixed Fe/W site distributed in the unit cell (Figure S1). Note that the Le Bail refinement in the columbite structure (3*a*, *b*, *c*; *Pbcn* space group) resulted in inferior profile fitting and worst GOF (goodness of fit). Consequently, the model in the small cell (*a*, *b*, *c*; *Pbcn* space group) was used to do the Rietveld refinement.

Atom	Wyck. pos.	*x*	*y*	*z*	SOF	U_{iso} (Å^2)
Fe1	4*c*	0	0.1654(5)	0.75	0.6667	0.0206(17)
W1	4*c*	0	0.1654(5)	0.75	0.3333	0.0206(17)
O1	8*d*	0.233(3)	0.144(2)	0.427(2)	1	0.012(4)

Table S1. Atomic coordinates and atomic displacement parameters of Fe_2WO_6 polyol sample at 800°C with *a* = 4.5995(15) Å, *b* = 5.6043(19) Å, *c* = 4.9718(19) Å, V = 128.16(8) Å^3, SG *Pbcn*.

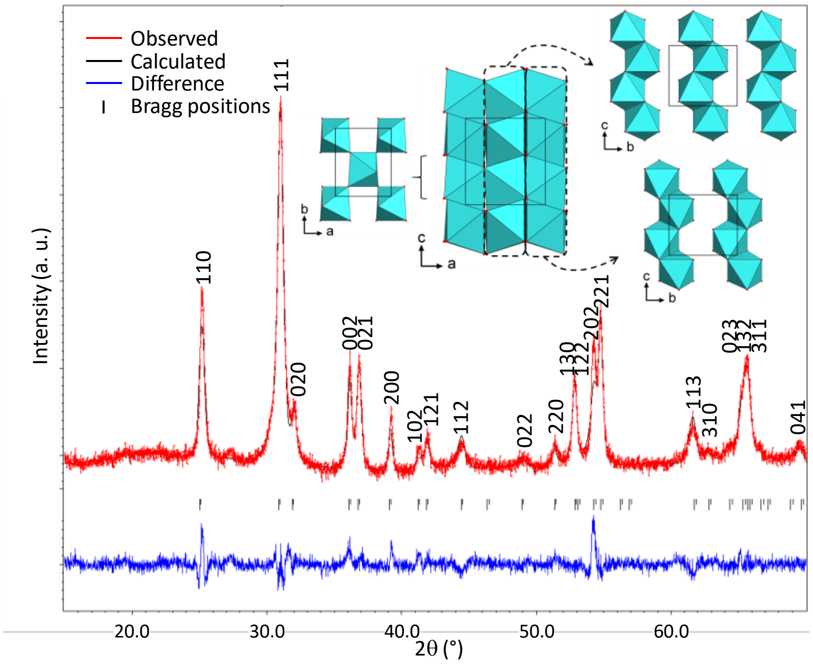

Figure S1. Final Rietveld refinement plot of the XRD data at 800°C of the Fe_2WO_6 polyol sample with Cu-$K\alpha1$-$K\alpha2$ radiation (R(obs) = 3.26 %, GOF = 1.33, wRp = 5.61 %). The inset shows the hypothetical structure of Fe_2WO_6 that can be described as the stacking along the *a* axis of layers made of zigzag chains of edge-sharing Fe/W octahedra shifted from one layer to another with a (1/2, 1/2, 0) vector. Layers are connected to each other through corner sharing octahedra.

2) Mössbauer experiments

^{57}Fe Mössbauer spectra were measured in transmission mode with a ^{57}CoRh source. During the measurements, both the source and the absorber were kept at ambient temperature (294 K). The spectrometer was operated in the constant acceleration mode with a triangular velocity waveform. The velocity scale was calibrated with the magnetically split sextet spectrum of a high-purity α-Fe reference absorber at room temperature. Since the fitting of the data is not possible using one doublet (one environment), they were fitted with an appropriate distribution of 6 Lorentzian profiles representing quadrupole doublets by least-squares methods using the program PC-Mos II[3]. In this way, spectral parameters such as the quadrupole splitting (QS), the isomer shift (IS), the linewidth at half maximum (LW) of the different spectral components were determined. Isomer shifts are given relative to the α-Fe.

Mössbauer spectra of the as prepared electrode, after being soaked in the electrolyte (blank) and after 10000 cycles are shown in figure S2, with the 6 Lorentzian profiles necessary, due to the distribution of the different Fe site environments. Figure S3 shows the Quadrupole Splitting distribution of the 6 contributions.

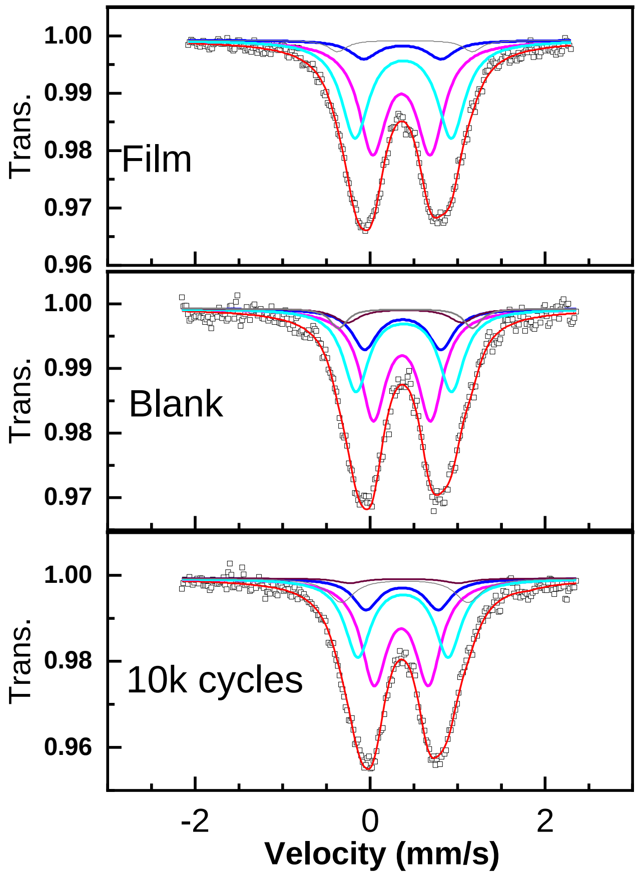

Figure S2. Room temperature ^{57}Fe Mössbauer spectra of all the samples showing the described in the text

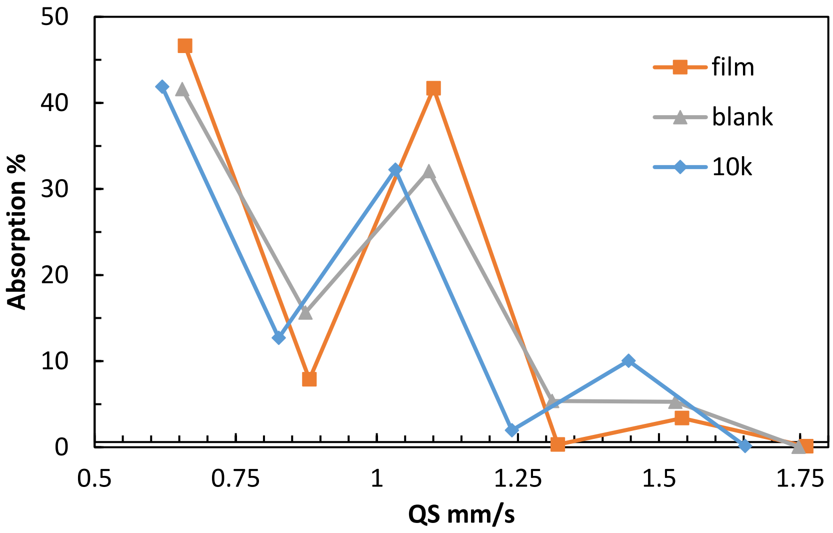

Figure S3. QS distribution for the three samples

Reference

1. Petříček, V.; Dušek, M.; Palatinus, L. Crystallographic Computing System JANA2006: General Features. *Zeitschrift für Kristallographie - Crystalline Materials* **2014**, 229, 345–352, doi:10.1515/zkri-2014-1737.
2. Guskos, N.; Sadlowski, L.; Typek, J.; Likodimos, V.; Gamari-Seale, H.; Bojanowski, B.; Wabia, M.; Walczak, J.; Rychlowska-Himmel, I. Magnetic and EPR Studies of α-, β-, and γ-Fe2WO6 Phases at Low Temperatures. Journal of Solid State Chemistry 1995, 120, 216–222, doi:10.1006/jssc.1995.1401.
3. Grosse, G. PC-Mos II, Version 1.0 Manual and Program Documentation; 1993 by FASTComTec, Oberhaching, Germany.
